# Are classifications of proximal radius fractures reproducible?

**DOI:** 10.1186/1471-2474-10-120

**Published:** 2009-10-01

**Authors:** Fabio T Matsunaga, Marcel JS Tamaoki, Eduardo F Cordeiro, Anderson Uehara, Marcos H Ikawa, Marcelo H Matsumoto, João BG dos Santos, João C Belloti

**Affiliations:** 1 Department of Orthopedics and Traumatology, Universidade Federal de São Paulo -- Escola Paulista de Medicina (Unifesp-EPM), São Paulo, Brazil; 2 Department Radiology, Universidade Federal de São Paulo -- Escola Paulista de Medicina (Unifesp-EPM), São Paulo, Brazil

## Abstract

**Background:**

Fractures of the proximal radius need to be classified in an appropriate and reproducible manner. The aim of this study was to assess the reliability of the three most widely used classification systems.

**Methods:**

Elbow radiographs images of patients with proximal radius fractures were classified according to Mason, Morrey, and Arbeitsgemeinschaft für osteosynthesefragen/Association for the Study of Internal Fixation (AO/ASIF) classifications by four observers with different experience with this subject to assess their intra- and inter-observer agreement. Each observer analyzed the images on three different occasions on a computer with numerical sequence randomly altered.

**Results:**

We found that intra-observer agreement of Mason and Morrey classifications were satisfactory (κ = 0.582 and 0.554, respectively), while the AO/ASIF classification had poor intra-observer agreement (κ = 0.483). Inter-observer agreement was higher in the Mason (κ = 0.429-0.560) and Morrey (κ = 0.319-0.487) classifications than in the AO/ASIF classification (κ = 0.250-0.478), which showed poor reliability.

**Conclusion:**

Inter- and intra-observer agreement of the Mason and Morey classifications showed overall satisfactory reliability when compared to the AO/ASIF system. The Mason classification is the most reliable system.

## Background

Fractures of the proximal radius are relatively common injuries, accounting for approximately 1/3 of all elbow fractures and about 1.7 to 5.4% of all fractures in adults [1,2]. Most of these (85%) occur in adults between 20 and 60 years of age (average of 30 to 40 years) with a male:female ratio of about 2:3 [1,2]. One third of the lesions are associated with others upper limb injuries, such as carpal bone fractures [3], distal radioulnar joint injuries [4,5], interosseous membrane injuries [6,7], capitellar fractures [8], and damage of the medial collateral ligament.

The most common mechanism of injury in these fractures is a fall onto an outstretched hand with an axial load on the radius. In such cases, the radial head and/or neck fracture when they collide with the capitellum, usually with forearm in pronation and the elbow in partial flexion [9-11].

Fractures of the proximal radius play an important role in injuries of the elbow, not so much by how frequently they occur, but mainly by the potential difficulties in treatment and the complications that can arise, sometimes with serious impairment of function due to pain and loss of mobility. Therefore, proper classification is essential in order to render the proper treatment.

Systems have been developed to help surgeons in classifying fractures into different and clinically useful groups for treatment definition.

In 1954, Mason described the first classification system dividing proximal radius fractures into three types. Type I fractures were nondisplaced or minimally displaced fractures of the head or neck; type II were displaced fractures (more than 2 mm) of the head or neck; and type III were severely comminuted fractures of the proximal radius [12]. In 1962, Johnston expanded the classification of Mason adding type IV, a fracture associated with dislocation of the elbow [13].

In 2008, van Riet and Morrey published a revision of the Mason classification, distinguishing between injuries associated with coronoid fractures, the olecranon fractures, and ligamentous injuries [14].

The AO/ASIF classification was created in 1986 and revised in 2007 [15]. It considers the seriousness of the bone injury and serves as a basis for treatment and prognosis.

The AO/ASIF system specifies three basic types: extra-articular, articular of the radius or ulna, and articular of the radius and ulna. With each group, the fractures are organized in increasing order of severity with regard to morphological complexity, difficulty in treatment, and prognosis. While this system is the most comprehensive, its intra- and inter-observer reliability has shown to be limited [16,17].

The purpose of a classification system is to name and describe the fractures according to their characteristics providing a hierarchy of those characteristics. A classification system should also guide action or intervention and assist in predicting outcomes of an intervention or treatment [18]. A good system needs to be valid, reliable, and reproducible. The perfect classification system should also standardize the language used to describe the fractures, offer guidelines for treatment, indicate the possibility of complications, and help determine the prognosis. The ideal system should also provide a mechanism to evaluate and compare the results with treatment of similar fractures treated at various centres and reported at different times in the literature [19].

Considering the need to classify the fractures of the proximal radius in an appropriate and reproducible manner, we sought to assess the reproducibility of the three most widely used classification systems. Thus we evaluated the intra- and inter-observer agreement of the Mason modified by Hotchkiss, Morrey and AO/ASIF classifications of proximal radius fractures.

## Methods

We analyzed 65 consecutive elbow radiographs performed on patients with fractures of the proximal radius. The patients were treated in the same hospital. Each of the 65 radiographs consisted of two views, anterior-posterior and lateral, and they were numbered, with the patients' names and ages concealed. Radiographs were excluded if the patient had incomplete skeletal development, pathologic fractures or previous elbow surgery.

The image quality was determined by two orthopaedic surgeons. The radiography was accepted only when both of these surgeons considered the radiographs acceptable.

Four observers familiar with the classification systems were selected for analysis. These observers were a second-year resident of orthopaedics (R2), a general orthopaedist (GO), a shoulder and elbow surgery specialist (SES), and a radiologist (RD).

To standardize the information for all observers, each were given self-explanatory diagrams with the classification systems. Each observer classified the 65 images at three different times, according to the three systems (Mason modified by Hotchkiss, Morrey and AO/ASIF). In the first evaluation (T1), the 65 digitized images of radiographs of each patient were viewed on a computer in numerical sequence. Three weeks later, in the second assessment (T2), the sequence of radiographs was randomly altered, as it was in the third assessment (T3), three weeks after T2. This sequence of randomization was known only by a person uninvolved in the assessment of the images.

The data were collected in spreadsheets and kappa coefficients were calculated for analysis according to the method proposed by Fleiss et al [20,21]. This method not only calculates the agreement expected by chance, as described earlier in the method of Scott and Cohen [22,23], but also the correlation among more than two observers in the evaluation of nominal variables. The kappa coefficient of agreement indicates the proportion of agreement among observers. The kappa values range from -1 to +1: values between -1 and 0 indicate that observed agreement was lower than that expected by chance, 0 indicates a level of agreement equal to that expected by chance, and +1 indicates total agreement. Overall, kappa values below 0.5 are considered unsatisfactory; values between 0.5 and 0.75 are considered satisfactory and appropriate, and values over 0.75 are considered excellent [24,25].

This project was approved by the Research Ethics Committee under No. 363/08, on April 4, 2008.

## Results

From the 65 initial radiographs six were excluded because of poor technical quality, leaving a sample size to 59 radiographs.

Table 1 summarizes the kappa values for intra-observer comparisons for each observer, at the three time points. The concordance was higher among the different with the Mason and Morrey classifications (mean κ = 0.582 and 0.554, respectively) than with the AO/ASIF classification (mean κ = 0.483).

**Table 1:** Kappa coefficients for intra-observer comparisons between the three time points (T1, T2 and T3).

**Observer**	**Classification**		
	**AO/ASIF**	**MORREY**	**MASON**

R2	0.676	0.666	0.710

GO	0.439	0.548	0.523

SES	0.305	0.356	0.409

RD	0.513	0.645	0.685

**Mean**	**0.483**	**0.554**	**0.582**

Observers: second-year resident of orthopaedics (R2), general orthopaedist (GO), shoulder and elbow surgery specialist (SES), radiologist (RD).

Table 2 shows the inter-observer kappa coefficients at each of the times of assessment. Inter-observer agreement was higher in the Mason (κ = 0.429-0.560) and Morrey (κ = 0.319-0.487) classifications than in the AO/ASIF classification (κ = 0.250-0.478).

**Table 2:** Kappa coefficients for inter-observer comparisons at each time point. (T1, T2, T3)

**Time Point**	**Classification**		
	**AO/ASIF**	**MORREY**	**MASON**

**T1**	0.250	0.319	0.429

**T2**	0.386	0.443	0.560

**T3**	0.478	0.487	0.551

## Discussion

The classification systems for this study were selected because they are the most commonly used and studied for fractures of the proximal radius [14]. Classification systems are of great importance in orthopaedic practice because they are used to describe fractures, guide treatment, and compare treatment outcome within and between studies in the literature. As a result, intra- and inter-observer concordances are essential for any classification system.

In the analysis of intra-observer agreement between three time points, the average kappa coefficient for the AO/ASIF classification was unsatisfactory (κ = 0.483), ranging from 0.305 (for the SES) to 0.676 (for the R2). The broad variability in these results is probably due to the complexity of the classification, and accord with the results of other studies that evaluated the classification for fractures of other bones [17,26]. Professional experience had no effect on intra-observer agreement with this system, as indicated by the highest kappa coefficient for the R2 and the lowest for the SES. For the Mason and Morrey classifications, intra-observer concordances were satisfactory and similar (κ of 0.692 and 0.644, respectively). The similarity of these coefficients was expected because the Morrey classification is derived from and more complex than the of Mason classification [14].

Regarding inter-observer concordances, the AO/ASIF rating was unsatisfactory at all times, although it improved with time, probably due to the learning curve. A similar pattern occurred with the Morrey classification, but the values were slightly higher but still unsatisfactory. The Mason classification had an unsatisfactory mean kappa coefficient at T1 (0.429). However, the means at T2 and T3 were satisfactory (0.560 and 0.551, respectively). These values may also be explained by the complexity of the classifications.

The AO/ASIF is a more complex system that involves the proximal radius as well as associated ulnar injuries and ligamentous injuries. These added variables will presumably have a negative impact on its reliability.

The results of this study were similar to those reported by Sheps et al. [27], which indicated that the correlation was unsatisfactory for the AO/ASIF classification and better for the system adapted by Hotchkiss from the Mason system. However, even this adapted Mason system was unsatisfactory when considering the lower limit of 95% of the confidence interval.

It is important to mention that the present study was limited to evaluating the agreement between the observers' opinions. This study was unable to measure the accuracy of each observer's opinion. To clarify the accuracy issue, studies in which clinical-radiographic diagnoses made by each observer compared with an examination result or a gold-standard procedure (i.e. one with high sensitivity and specificity) would be needed in order to prove the proposed diagnosis.

We have made the first step to the development of the ideal classification once we studied the reliability of the most widely used classification systems. In the continuing search for this ideal classification there is a need to perform new prospective studies with large number of patients to determine which variables (displacement of the fragments, intra-articular/metaphyseal comminution, associated injuries, patient's age) can guide the ideal treatment and predict the prognosis of such fractures through radiographic examination.

## Conclusion

Inter- and intra-observer agreement of the Mason and Morey classifications showed overall satisfactory reliability when compared to the AO/ASIF system.

The Mason classification is the most reliable system.

## Competing interests

The authors declare that they have no competing interests.

## Authors' contributions

FTM participated in the design of the study, collected, digitalized and randomized the radiographs, helped to draft the manuscript, revised the bibliography for references and performed the statistical analysis; MJST conceived the study design, analyzed the radiographs and classified the fractures, helped to draft the manuscript and revised the bibliography for references; EFC participated in the design of the study, analyzed the radiographs and classified the fractures and translated the manuscript to English; AU: participated in the design of the study, analyzed the radiographs and classified the fractures; MHI participated in the design of the study, analyzed the radiographs and classified the fractures; MHM conceived the study design, revised the article and gave the final approval of the version to be published; JBGS participated in the design of the study, revised the article and gave the final approval of the version to be published; JCB participated in the design of the study, revised the article; FF revised the article and gave the final approval of the version to be published. All authors read and approved the final manuscript.

## About the authors

Fabio T Matsunaga^1^, Marcel J S Tamaoki^2^, Eduardo F Cordeiro^3^, Anderson Uehara^4^, Marcos H Ikawa^5^, Marcelo H Matsumoto^6^, João B G dos Santos^7^, João C Belloti^8^.

^1^Second-year resident in the Department of Orthopedics and Traumatology, Universidade Federal de São Paulo -- Escola Paulista de Medicina (Unifesp-EPM), São Paulo, Brazil.

^2^Attending physician in Shoulder and Elbow Surgery Sector in the Department of Orthopedics and Traumatology, Universidade Federal de São Paulo -- Escola Paulista de Medicina (Unifesp-EPM), São Paulo, Brazil.

^3^Second-year resident in the Department of Orthopedics and Traumatology, Universidade Federal de São Paulo -- Escola Paulista de Medicina (Unifesp-EPM), São Paulo, Brazil.

^4^Attending physician in Shoulder and Elbow Surgery Sector in the Department of Orthopedics and Traumatology, Universidade Federal de São Paulo -- Escola Paulista de Medicina (Unifesp-EPM), São Paulo, Brazil.

^5^Attending physician in the Department Radiology, Universidade Federal de São Paulo -- Escola Paulista de Medicina (Unifesp-EPM), São Paulo, Brazil.

^6^Head of the Shoulder and Elbow Surgery Sector, Department of Orthopedics and Traumatology, Universidade Federal de São Paulo -- Escola Paulista de Medicina (Unifesp-EPM), São Paulo, Brazil.

^7^Adjunct professor and head of Hand Surgery Clinic, Department of Orthopedics and Traumatology, Universidade Federal de São Paulo -- Escola Paulista de Medicina (Unifesp-EPM), São Paulo, Brazil.

^8^Attending physician in Hand Surgery Clinic, Department of Orthopedics and Traumatology, Universidade Federal de São Paulo -- Escola Paulista de Medicina (Unifesp-EPM), São Paulo, Brazil.

## Pre-publication history

The pre-publication history for this paper can be accessed here:


